# An Array of MOX Sensors and ANNs to Assess Grated Parmigiano Reggiano Cheese Packs’ Compliance with CFPR Guidelines

**DOI:** 10.3390/bios10050047

**Published:** 2020-05-02

**Authors:** Marco Abbatangelo, Estefanía Núñez-Carmona, Veronica Sberveglieri, Dario Zappa, Elisabetta Comini, Giorgio Sberveglieri

**Affiliations:** 1 Department of Information Engineering, University of Brescia, Brescia, via Branze, 38, 25123 Brescia, BS, Italy; dario.zappa@unibs.it (D.Z.); elisabetta.comini@unibs.it (E.C.); giorgio.sberveglieri@unibs.it (G.S.); 2 CNR-IBBR, Institute of Bioscience and Bioresources, via Madonna del Piano, 10, 50019 Sesto Fiorentino, FI, Italy; estefania.nunezcarmona@ibbr.cnr.it (E.N.-C.); veronica.sberveglieri@ibbr.cnr.it (V.S.); 3 NANO SENSOR SYSTEMS, NASYS Spin-Off University of Brescia, Brescia, via Camillo Brozzoni, 9, 25125 Brescia, BS, Italy

**Keywords:** electronic nose, nanowire gas sensors, MOX sensors, rapid detection, food quality control, Parmigiano Reggiano, artificial neural network

## Abstract

Parmigiano Reggiano cheese is one of the most appreciated Italian foods on account of its high nutrient content and taste. Due to its high cost, these characteristics make this product subject to counterfeiting in different forms. In this study, an approach based on an array of gas sensors has been employed to assess if it was possible to distinguish different samples based on their aroma. Samples were characterized in terms of rind percentage, seasoning, and rind working process. From the responses of the sensors, five features were extracted and the capability of these parameters to recognize target classes was tested with statistical analysis. Hence, the performance of the sensors’ array was quantified using artificial neural networks. To simplify the problem, a hierarchical approach has been used: three steps of classification were performed, and in each step one parameter of the grated cheese was identified (firstly, seasoning; secondly, rind working process; finally, rind percentage). The accuracies ranged from 88.24% to 100%.

## 1. Introduction

Among all the typical Italian foods, Parmigiano Reggiano (PR) cheese is one of the most famous products. It is well-known worldwide since 40% of the total value of sales is represented by exports [1], even if consumers are not always aware of the differences between PR and the other parmesan-like cheeses [2]. In terms of commercial importance, PR is the most important Protected Designation of Origin (PDO) Italian cheese [3]. Its production is regulated by the Parmigiano Reggiano Cheese Consortium (CFPR). According to European Regulation 510/2006, PDO can be exclusively assigned to cheeses made with a traditional established production technology in a restricted area of Italy (the provinces of Parma, Reggio Emilia, Modena, Mantova, and Bologna) and with milk produced in the same area [4].

PR is also one of the most counterfeited foods since it is an appreciated cheese, but it has a high cost if compared to similar hard cheeses. Indeed, fraud rates of between 20% and 40% have been estimated, the latter predominantly in the grated form [5]. In particular, this second data point is important because it was the starting point of this work and the previous one [6]. Indeed, for grated PR cheese, some rules established by the CFPR must be followed to bear the brand of PR on the market. Among all the parameters, those that have been taken into account in our research are the ripening (at least 12 months) and the rind percentage that cannot be over 18% w/w compared to pulp [7]. Since rind is a cheaper and less esteemed material than pulp, frauds where there is more than 18% rind in the packs sold on the market can happen.

In this work, an electronic nose-like device (called S3) has been used to analyze rind in grated PR cheese samples through the emission of volatile organic compounds (VOCs). Electronic noses are systems that can contain different types of chemical gas sensors. In recent years, this kind of device has received considerable attention for its potentialities in various fields, with excellent results [8,9,10,11,12,13]. Furthermore, compared with such classical chemical techniques as gas chromatography (GC), they are more rapid, cheaper, more portable, and more user-friendly. Conversely, the main disadvantage is that they are less precise. Indeed, sensors arrays are not able to give the name of the molecules they are analyzing as output; however, they can be trained to recognize target samples thanks to machine learning algorithms.

Many types of research have been carried out regarding dairy products, from milk to seasoned cheese [14]. It has been demonstrated that it is possible to recognize unspoiled milk from milk containing a concentration of bacteria potentially unsafe for human health, using conducting polymer sensors [15]; potentially, sensors can follow bacteria growth in milk and its shelf-life with metal oxide (MOX) semiconductor sensors [16]; there is the possibility of distinguishing natural milk flavorings from synthetic ones with MOX sensors [17]; finally, they can be used to evaluate the effects of feed supplementations to cows’ diets on their milk [18]. As regards cheese distinction, many works summarize the employment of electronic noses and compare their performance with other techniques [19,20,21]. Gursoy et al. showed that an ion mobility based electronic nose system called “MGD-1” can recognize different seasoning stages of Emmental and different countries of origin of the same cheese [22]; in 2011, Cevoli et al. used MOX sensors to classify Pecorino cheese according to its ripening time [23]; piezoelectric quartz crystals were employed by Pais et al. to recognize cheeses made of different kinds of milk and textures [24]; finally, the same types of sensors were used by Valente et al. to distinguish cheeses made of raw milk from those prepared with pasteurized milk [25]. These examples show how devices of this kind have the potential to be used to evaluate food quality and identity and to prevent fraud.

This research aimed to generalize the results obtained in Reference [6]. Indeed, we used a methodology similar to the one tuned in the previous work on a larger set of samples with new parameters and optimized the training of the system to assess whether grated PR parameters were in compliance with the guidelines. Furthermore, based on the results of the cited work, only artificial neural networks (ANNs) were used for the aforementioned purpose.

## 2. Materials and Methods

### 2.1. Samples Under Analysis

Analyzed samples were packaged under vacuum at the headquarters of the CFPR. They came from two different ripening stages: 12 and 24 months. For each of these, 11 different combinations of pulp–rind were prepared (expressed in rind percentage): 0%, 5%, 16%, 18%, 20%, 26%, 32%, 45%, 55%, 63% and 100%. These percentages were grouped into three classes: rind lower than 18% (guideline limit), between 19% and 26%, higher than 26%. Furthermore, two kinds of rind working processes were considered: washed-rind (WR) and scraped-rind (SR). These are standard processes done to clean the outside of the cheese wheel. From each sample, 14 replicas were prepared; it must be considered that the category “0% rind” was characterized by only two kinds of samples (12 months and 24 months ripening), while the other percentages had four kinds of samples, two for each ripening stage depending on the rind working process. Furthermore, due to some technical inconveniences, some measures were discarded since they were incomplete. Hence, the total number of replicas obtained from the samples and analyzed was equal to 452. The number of replicas divided for the three parameters is shown in Table 1. The sum of the number of replicas for the rind working process is different from the total of the respective seasoning. Missing replicas correspond to 0% of samples since they do not contain rind. For this reason, 14 replicas belonging to 0%-12 months seasoned specimens and 12 belonging to 0%-24 months are not reported in the table. Samples were stored at 4 °C until the moment when they were prepared for analysis. An amount of 2 g of grated cheese was positioned in 20 mL glass headspace vials sealed by a metal cap with a PTFE-silicon membrane.

### 2.2. S3 Analysis

The small sensors system (S3) device used in the present work has been completely designed and constructed at SENSOR Laboratory (University of Brescia, Brescia, Italy) in collaboration with NASYS S.r.l., a spin-off of the University of Brescia. It has been used in many applications [26,27,28]. Being an electronic nose-like device, the S3 comprises MOX gas sensors as sensing elements; in this case, the number of sensors was 8. Three of them were in the nanowire form [29,30,31,32]: Two were tin oxide nanowire sensors, both grown through the vapor-liquid-solid technique [33] (one of them functionalized with gold clusters); the third sensor had a sensing layer of copper oxide nanowires. The three sensors were prepared using the rheotaxial growth and thermal oxidation (RGTO) technique: One of them SnO_2_ functionalized with gold clusters, while the other two were pure tin oxide and worked at different operating temperatures. Details of S3 sensors made at SENSOR Laboratory are summarized in Table 2. The last two were commercial MOX from Figaro Engineering Inc. (Osaka, Japan): TGS2611 and TGS2602, which are sensitive to natural gases and odorous gases like ammonia, respectively, according to the datasheet of the company.

S3 analyzed the VOCs present in the headspace of the samples. The volatile fraction was then aspirated and transported to the sensors’ chamber to be analyzed. To avoid any influence of the surrounding environment to the sensors’ response, the chamber was isolated. Furthermore, the sensors’ baseline was obtained by filtering the air of the surrounding environment with a small metal cylinder (21.5 cm in length, 5 cm in diameter) filled with activated carbons.

The instrument was also provided with the auto-sampler headspace system HT2010H (Hta S.r.l., Brescia, Italy), supporting a 42-loading-sites carousel. The vials were placed in a randomized mode into the carousel. Each vial was incubated at 50 °C for 5 min in the auto-sampler oven and shaken for 1 min during the incubation. The sample headspace was flushed at 80 sccm into the sensing chamber for 1 min (analysis time).

### 2.3. Data Analysis

Data analysis was performed using MATLAB^®^ R2019b software (MathWorks, Natick, MA, USA). Sensors’ responses in terms of resistance (Ω) were normalized to the first value of the acquisition (R_0_). For all of the sensors, five different features were extracted. In fact, unlike the previous work, the increase in the number of samples and variables considered has led to the search for new features to keep the recognition rate of the system high. Firstly, the difference between the first value and the minimum value during the analysis time was calculated. Hence, the ΔR/R_0_ parameter was calculated. Secondly, the minimum and the maximum of the first derivative values were found for n-type sensors and p-type sensor respectively. The following two features regarded the area under the signal that was calculated in two ways: Considering (a) the time axis from the initial value up to the maximum/minimum value of the signal and (b) all of the sensors’ responses. Finally, the fall time (for n-type sensors) and the rise time (p-type sensor) were calculated, considering 10% and 90% as the reference levels.

Features were checked using ANOVA and multiple comparison procedures with Tukey’s honestly significant difference procedure [34] to assess the ones capable of distinguishing among the target classes. Only the features of which the class averages were significantly different were selected. Once the optimal subset of features was identified, an ANN was trained. The classification task was divided into three steps to enhance the performance of the networks. As described in Reference [6], a hierarchical approach has been followed to simplify models for classification, since the combination of parameters of grated PR cheese used in this study was equal to 15. In the first step, the ANN was trained on the recognition of seasoning degree; in the second step, for each seasoning state the different working process was individuated; finally, the rind percentage was detected. The ANN’s structure changed depending on the dimension of the chosen subset of features done at each step: in general, from one to three hidden layers with the same activation function that is hyperbolic tangent sigmoid and one output layer with the competitive transfer function. The different dimensions of the subset reflect the number of neurons in the layers, too. Results are provided in terms of percentage of accuracy.

## 3. S3 Analysis: Results and Discussion

**Figure 1:**
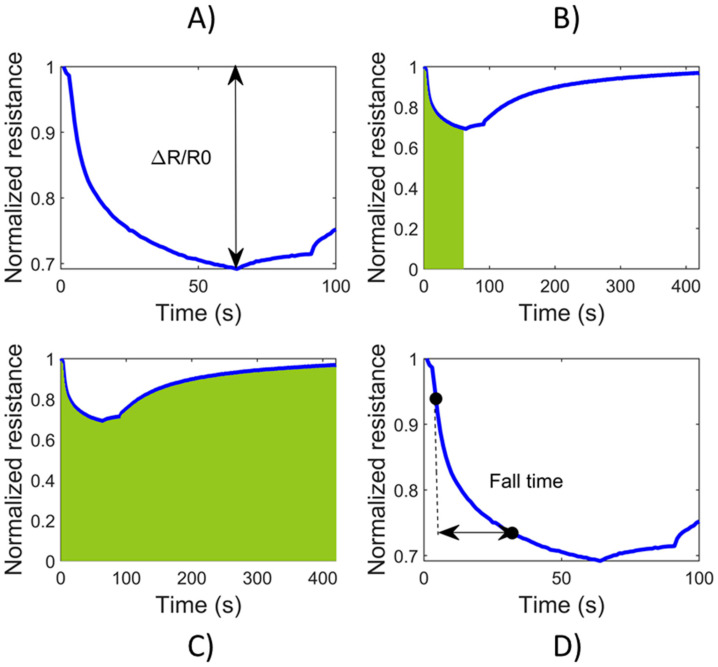
The four features extracted from the normalized signal of nanowire SnO_2_ sensor: (**A**) the variation of resistance from the baseline (ΔR/R_0_); (**B**) the area under the signal up to the minimum value (in green); (**C**) the total area under the signal (in green); (**D**) the fall time between the levels of 10% and 90%.

The analysis of S3 signals started with the visualization of the sensors’ response to samples. The signal trend of the nanowire SnO_2_Au sensor (n-type) is shown in Figure 1. Furthermore, the four features extracted from the signals are indicated. For the p-type CuO-based sensor, the same procedure has been followed: exchanging the minimum values involved with the maximum ones, and the fall time with the rise time of the signal.

**Figure 2:**
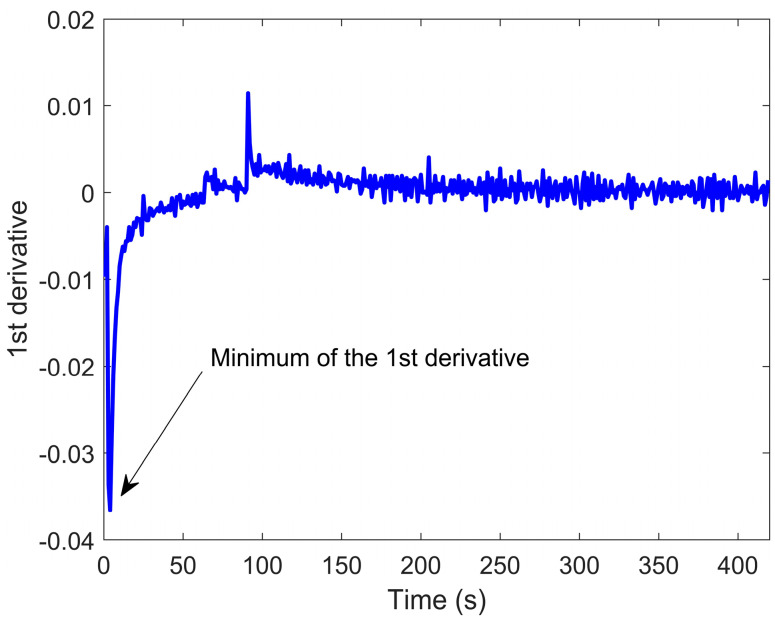
The fifth feature has been extracted from the first derivative of the signal and is the minimum value of the derivative itself.

In Figure 2, the first derivative of a nanowire SnO_2_Au sensor is reported. The signal is characterized by two spikes: the former has been chosen as a feature since it could give information about the speed of resistance change after the sensor was exposed to VOCs; the latter is due to the switch from the sample’s headspace to filtered ambient air, but it has not been considered for the following analysis.

**Figure 3:**
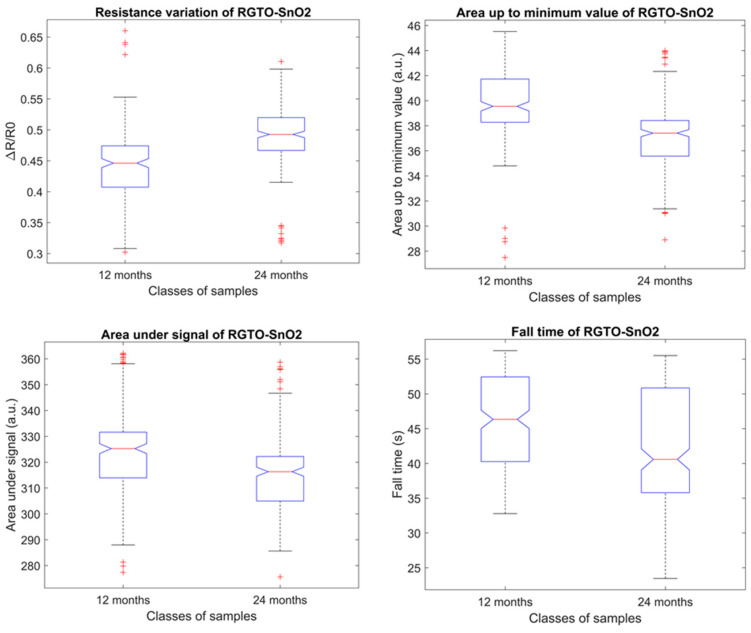
Boxplots of the four features selected in the first step for the RGTO SnO_2_ (400 °C) sensor. The median value of the distribution is displayed in red; the red ‘+’ indicates outliers for that distribution.

In Figure 3, the boxplots of features selected to train ANNs in the first step of the mentioned approach are shown. Since out of the forty features, twenty-two were selected through the Tukey’s honestly significant difference procedure, it has been decided to display boxplots of one RGTO SnO_2_ as a general indication. Even so, four of the five different typologies of features are represented. The idea behind the selection of the features in the other steps remains the same, hence the relative boxplots are not reported here.

In the boxplot, the red central line indicates the median of the distribution, while the red ‘+’ symbols reveal the outliers. The notch of the plot displays the confidence interval around the median value. According to [35], even if it is not a formal test, if the notches of two boxes do not overlap it is possible to assume that there is strong evidence that the two medians differ, with a confidence level of 95%. This is the case in the presented boxplots; this assumption has been confirmed by the Tukey’s test. The plots reveal that the variation of the normalized resistance is lower for the 12 months samples compared to the 24 months samples. This result was expected since during the aging process the aroma of PR increases due to the activity of endogenous microorganisms that change organoleptic and biochemical characteristics [36,37]. Consequently, it is not surprising to find that the values of the two areas calculated are higher for the 12 months specimens. Furthermore, an additional indicator of the lesser intensity of 12 months PR aroma is the fall time: indeed, there is a difference of about 5 s between the two classes, showing that the reactions that take place over the sensing layer are lower for the less seasoned cheese.

In Table 3, the parameters that have been chosen for the classification process at each step are stated. Each sensor contributes in a different way to the recognition of the parameters. It is interesting to notice the difference between the two RGTO SnO_2_ sensors heated at two working temperatures. The sensor with the lower temperature, i.e., 300 °C, did not provide any useful information for the recognition of the seasoning degree and only three parameters in the other two steps. Conversely, for the RGTO at 400 °C, the ΔR/R_0_ and area parameters have been selected at each step for the classification of the samples. It is reasonable to think that the higher working temperature enhances the sensitivity towards the VOCs of the different samples. Finally, it can be seen in the table that the most frequently selected features, in descending order, are the area up to min/max value, ΔR/R_0_, total area, fall time/rise time, and min/max value of the 1st derivative.

In Table 4, the accuracy at each step of the ANNs is shown; the accuracies of the previous work are reported in brackets to make comparison easier. To train the networks, the whole dataset was divided into a training set and test set with a 2:1 ratio at each step. The accuracies in the table are calculated on the test set. Comparing the results with the previous work [6], it is possible to notice that some percentages of classification are lower. It is because the complexity of the faced task was increased since the newly-analyzed samples were characterized by new values of rind percentage. The reduction of accuracy concerns the first two steps, passing from 100% to the value listed in Table 4, and it can be considered a physiological result, having tripled the number of replicas to build the ANNs. Conversely, as regards step 3, the classification rate is higher compared to the previous analysis, especially the recognition of rind percentage in WR samples: indeed, it rises from 63.8% and 58.8% to 88.24% and 96.96%, respectively. This enhancement could be due to the increase of information obtained from the sensors, passing from one type of feature (ΔR/R_0_) to five. Furthermore, the different number of features employed at each step and the consequent architecture of the ANNs made sure that each step had an optimized model that differed from the others.

## 4. Conclusions

This study aimed to confirm and to generalize the results obtained in previous work. The task of distinguishing grated PR with different rind percentages with an array of gas sensors has been expanded, adding other classes for the rind percentage parameter. The hierarchical approach proposed in the previous work was used and ANNs were employed for the classification task. The ANNs’ classification rates were high during each step, ranging from 88.24% to 100%. It was possible to obtain these results thanks to the increase in information extracted by the sensors’ signals and the different models of ANNs created. Furthermore, they confirm and generalize the results shown in the previous paper. Indeed, in the face of a slight decrease in accuracy in the first two steps, there has been a substantial increase in the third step. In conclusion, the potentiality of the system to give a fast response to the recognition of grated PR cheese has been validated.

## Figures and Tables

**Table 1:** List of the analyzed samples and number of replicas, divided according to rind percentage, ripening, and rind working process.

Seasoning	N° of Replicas	Rind Working Process	N° of Replicas	Rind Percentage	N° of Replicas
12 months	211	WR	105	≤18%	23
				19–26%	21
				>26%	61
		SR	92	≤18%	26
				19–26%	24
				>26%	42
24 months	241	WR	103	≤18%	36
				19–26%	26
				>26%	41
		SR	126	≤18%	38
				19–26%	23
				>26%	65

**Table 2:** Type, composition, morphology, and operating temperature for S3 sensors made at the SENSOR Laboratory.

Material (Type)	Composition	Morphology	Operating Temperature (°C)
SnO_2_Au (n)	SnO_2_ functionalized with Au clusters	RGTO	400 °C
SnO_2_ (n)	SnO_2_	RGTO	300 °C
SnO_2_ (n)	SnO_2_	RGTO	400 °C
SnO_2_Au (n)	SnO_2_ grown with Au and functionalized with gold clusters	Nanowire	350 °C
SnO_2_ (n)	SnO_2_ grown with Au	Nanowire	350 °C
CuO (p)	CuO	Nanowire	400 °C

**Table 3:** List of the features selected at each step of the analysis.

Sensor	Feature Selected in Step 1	Feature Selected in Step 2	Feature Selected in Step 3
RGTO SnO_2_ (300 °C)	-	Min value 1st derivative Area up to min value	Min value 1st derivative
Nanowire SnO_2_Au	ΔR/R_0_ Area up to min value Total area Fall time	ΔR/R_0_ Min value 1st derivative Area up to min value Total area	ΔR/R_0_ Min value 1st derivative Area up to min value
Nanowire SnO_2_	ΔR/R_0_ Area up to min value Total area Fall time	Area up to min value	ΔR/R_0_ Area up to min value Fall time
CuO	ΔR/R_0_ Area up to the max value Total area Rise time	Max value 1st derivative	ΔR/R_0_ Max value 1st derivative Area up to the max value Total area
TGS2611	ΔR/R_0_	ΔR/R_0_ Area up to min value	Total area Fall time
TGS2602	ΔR/R_0_ Area up to min value Total area Fall time	Area up to min value Fall time	ΔR/R_0_
RGTO SnO_2_ (400 °C)	ΔR/R_0_ Area up to min value Total area Fall time	Fall time	ΔR/R_0_ Area up to min value Total area
RGTO SnO_2_Au	Min value 1st derivative	Min value 1st derivative	ΔR/R_0_ Area up to min value Total area

**Table 4:** Classification rates in the percentage of ANNs for each step. In brackets, the results of the previous work are reported.

Step 1	Classification Rate	Step 2	Classification Rate	Step 3	Classification Rate
Seasoning	98.66% (100%)	12 months working process	98.55% (100%)	12 months WR rind percentage	88.24% (63.8%)
				12 months SR rind percentage	100% (96.1%)
		24 months working process	91.14% (100%)	24 months WR rind percentage	96.97% (58.8%)
				24 months SR rind percentage	100% (100%)
